# Multivariate comparison of taxonomic, chemical and operational data from 80 different full-scale anaerobic digester-related systems

**DOI:** 10.1186/s13068-024-02525-1

**Published:** 2024-06-20

**Authors:** Pascal Otto, Roser Puchol-Royo, Asier Ortega-Legarreta, Kristie Tanner, Jeroen Tideman, Sjoerd-Jan de Vries, Javier Pascual, Manuel Porcar, Adriel Latorre-Pérez, Christian Abendroth

**Affiliations:** 1 Institute of Waste Management and Circular Economy, Technische Universität Dresden, Pirna, Germany; 2 Darwin Bioprospecting Excellence, S.L. Parc Cientific Universitat de Valencia, Paterna, Valencia Spain; 3 Bioclear Earth B.V., Groningen, The Netherlands; 4 Institute for Integrative Systems Biology I2SysBio, (University of Valencia - CSIC), Paterna, Spain; 5 Chair of Circular Economy, Brandenburgische Technische Universität Cottbus-Senftenberg, Lehrgebäude 4A R2.25, Siemens-Halske-Ring 8, 03046 Cottbus, Germany

**Keywords:** Anaerobic digestion, 16S rRNA sequencing, Microbiome, Core microbiome, Multivariate analysis, Parameter dependency

## Abstract

**Background:**

The holistic characterization of different microbiomes in anaerobic digestion (AD) systems can contribute to a better understanding of these systems and provide starting points for bioengineering. The present study investigates the microbiome of 80 European full-scale AD systems. Operational, chemical and taxonomic data were thoroughly collected, analysed and correlated to identify the main drivers of AD processes.

**Results:**

The present study describes chemical and operational parameters for a broad spectrum of different AD systems. With this data, Spearman correlation and differential abundance analyses were applied to narrow down the role of the individual microorganisms detected. The authors succeeded in further limiting the number of microorganisms in the core microbiome for a broad range of AD systems. Based on 16S rRNA gene amplicon sequencing, MBA03, *Proteiniphilum*, a member of the family *Dethiobacteraceae*, the genus *Caldicoprobacter* and the methanogen *Methanosarcina* were the most prevalent and abundant organisms identified in all digesters analysed. High ratios for *Methanoculleus* are often described for agricultural co-digesters. Therefore, it is remarkable that *Methanosarcina* was surprisingly high in several digesters reaching ratios up to 47.2%. The various statistical analyses revealed that the microorganisms grouped according to different patterns. A purely taxonomic correlation enabled a distinction between an acetoclastic cluster and a hydrogenotrophic one. However, in the multivariate analysis with chemical parameters, the main clusters corresponded to hydrolytic and acidogenic microorganisms, with SAOB bacteria being particularly important in the second group. Including operational parameters resulted in digester-type specific grouping of microbes. Those with separate acidification stood out among the many reactor types due to their unexpected behaviour. Despite maximizing the organic loading rate in the hydrolytic pretreatments, these stages turned into extremely robust methane production units.

**Conclusions:**

From 80 different AD systems, one of the most holistic data sets is provided. A very distinct formation of microbial clusters was discovered, depending on whether taxonomic, chemical or operational parameters were combined. The microorganisms in the individual clusters were strongly dependent on the respective reference parameters.

**Graphical Abstract:**

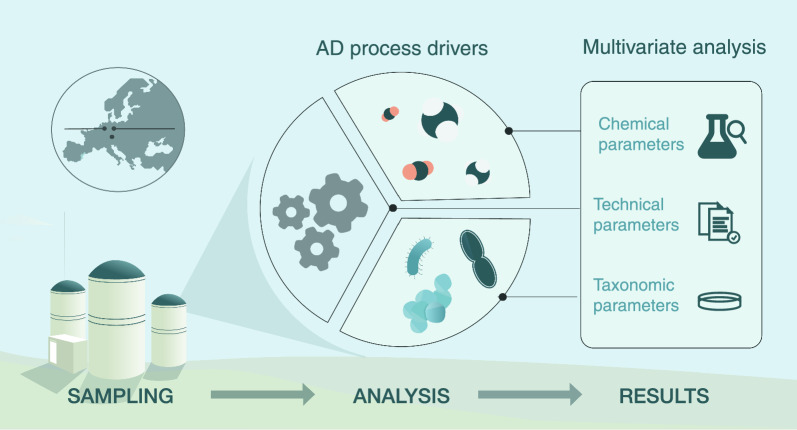

**Supplementary Information:**

The online version contains supplementary material available at 10.1186/s13068-024-02525-1.

## Introduction

The production of biogas through anaerobic digestion (AD) can contribute to achieving key sustainability objectives such as the development of a circular economy, the transition to a bio-economy and the independent production of renewable electricity and gas [1]. Consequently, secondary raw materials such as organic waste, municipal sewage sludge, green waste, animal excrements, and agricultural residues can be converted into valuable resources such as methane and fertilizer. The conversion of these various organic materials into biogas is essentially carried out by complex microbial communities that constitute the AD microbiome [2, 3]. Understanding the microorganisms involved, the conditions in which they occur, and the operational and chemical factors that influence them, provides an opportunity to make the process more robust and efficient. AD is divided into four stages, each of which is carried out by different consortia of microorganisms with different environmental requirements [4]. In the first phase, hydrolysis, complex organic materials such as proteins, fats, and carbohydrates are hydrolysed into the corresponding monomers and oligomers. These are then metabolized in the second phase, acidogenesis, to intermediates such as propionate, butyrate, other short-chain volatile fatty acids (VFAs), and alcohols. In the third phase, known as acetogenesis, acetic acid, CO_2_ and H_2_ are obtained by fermenting VFAs. Subsequently, these products are converted to CH_4_ and CO_2_ in a final phase, methanogenesis [2, 4]. The microbial communities of these four phases are generally categorized as fermentative bacteria, methanogenic archaea, and syntrophic bacteria. Fermentative communities carry out the first three phases, while methanogenic archaea use the intermediate products of the third phase to produce CH_4_ and CO_2_ in the fourth phase. Syntrophic communities are composed of microorganisms that work together in a mutualistic relationship [5].

The main added value of studying a broad range of AD microbiomes is to make general statements about the chemical, taxonomic and operational factors influencing them. As a result, AD processes can be better bioengineered in the future to increase the efficiency, speed, robustness and adaptability of AD systems [6]. Culture-independent methods such as 16S rRNA gene or metagenomics sequencing assist with the complete characterization of the microbiome [7]. Analysis of 16S rRNA gene amplicons has been used in several studies to investigate microbial communities in AD systems [8, 9]. Correlations between microbial profiles and various operational or physicochemical operating parameters were established to assign changes in microbial community structure. In particular, studies of the microorganisms that are always present in AD systems, i.e. the core microbiome, are promising. However, current studies are lacking in terms of the diversity of AD systems sampled and the completeness of the metadata available. Although several studies have provided insights into the complexity of the underlying microbiome communities, it is not clear how many microorganisms are involved in AD, what tasks the individual microorganisms fulfil in this process and what they depend on. Puig-Castellví et al. found 1145 operative taxonomic units (OTUs) in a reactor that was co-digesting wastewater sludge [10]. Calusinska et al. observed 20 industrial biogas plants and confirmed the hypothesis that different systems occur with different core microbiomes [11]. Kirkegaard et al. compared nine large-scale fermenters and looked for microbes that were present in all of them. This allowed the authors to narrow down the core microbiome in these fermenters to 300 species, which accounted for 80% of all reads [12]. As a result, researchers are increasingly using multivariate analyses to compare a large number of operational and physicochemical parameters with large DNA-based data sets [2, 13]. A particularly comprehensive study on this topic was conducted by Hassa et al., comparing 67 full-scale digesters from 49 similar agricultural plants. This study on similar agricultural AD systems shows the presence of microbial indicators for certain process conditions such as temperature, ammonia and substrate selection [14]. In summary, individual studies on the core microbiome and the chemical and/or operational factors influencing the taxonomy have been carried out for wastewater treatment plants [15, 16], agricultural systems [2, 14], AD systems fed with organic food waste [17, 18] and AD systems fed with animal excrement [19, 20]. Similar to [69], studies that combine such a diversity of AD systems with a complete chemical, taxonomic and operational data set are required to make generalized statements. The present study fills this gap as it contributes to the understanding of the microbial ecology of a wide range of AD systems and their interaction with the operating conditions of the system. The results are validated by one of the largest data sets, which includes microbial, chemical and operational data from 80 different AD systems. The obtained results extend the basic understanding of the microbiome in AD, highlight the key microbial players in the process and analyse how different variables influence the underlying microbial communities.

## Materials and methods

### Sample selection and collection

**Fig. 1:**
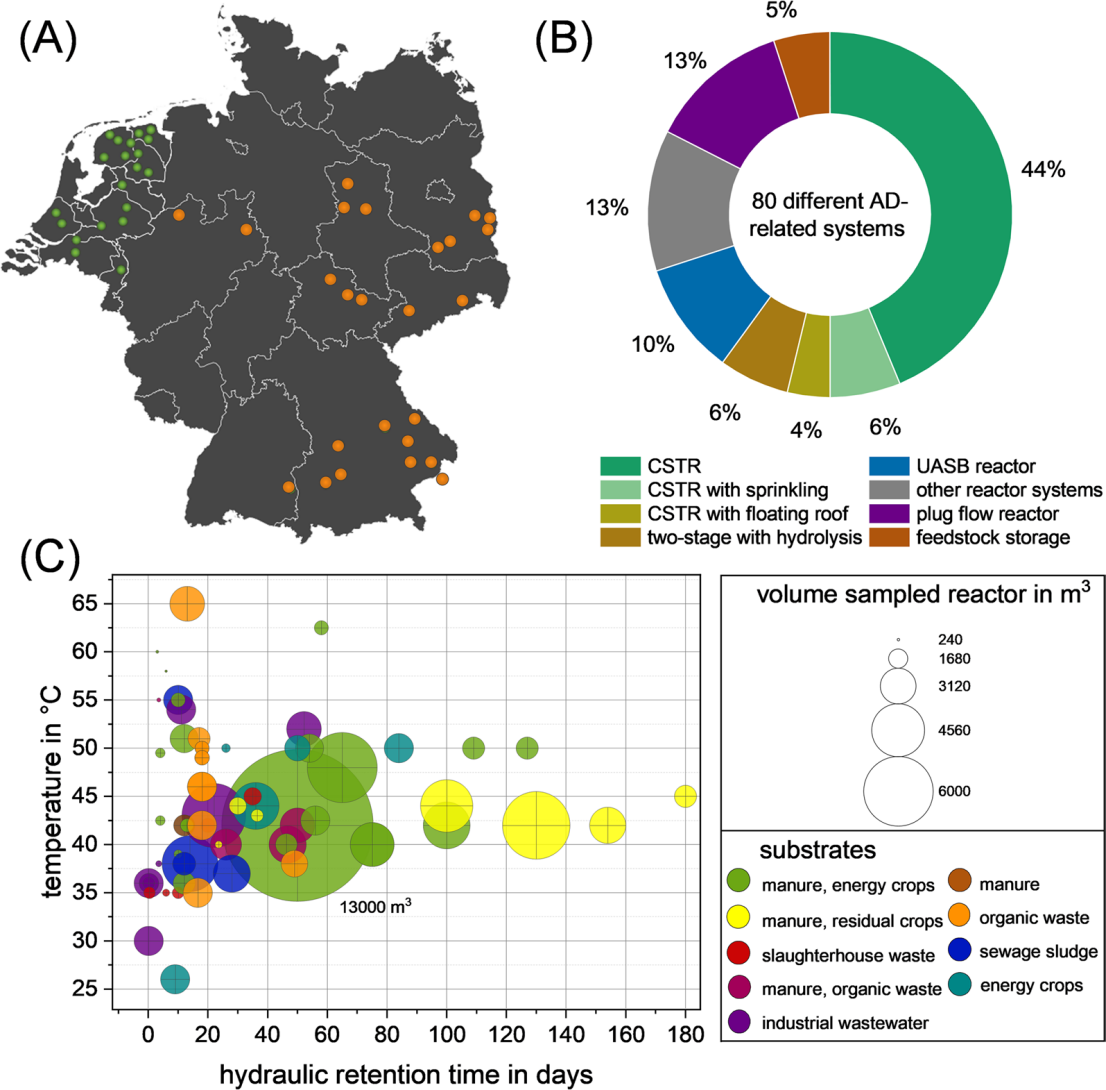
Overview of all sampled AD systems. **A** Location of AD systems sampled in Germany (orange) and the Netherlands (green). **B** Types of AD systems sampled. **C** Main operational data: temperature (y-axis) and the average retention time (x-axis) are shown. The bubble size indicates the reactor size and the colours indicate the main substrates in the respective feedstock

A comprehensive set of 80 samples was taken at 45 sites from different full-scale anaerobic digestion plants. These reactors have been specially selected to represent a variety of different reactor systems, feedstock compositions and operational conditions such as temperature. Sampling took place from August to December 2021. A total of 80 distinct digester samples were collected in Germany, Austria and the Netherlands (Fig. 1A). The Technische Universität Dresden collected the samples in Germany and Austria, whereas the samples from the Netherlands were collected by Bioclear Earth B.V. In both cases, the sampling procedure was based on the exact same protocol. Samples for chemical analysis were collected in sterile 1-l sampling bottles. Samples for metagenomic DNA extraction and sequencing were collected in sterile 50-ml Falcon tubes. In order to preserve the DNA and prevent microbial changes, the samples were mixed 1:1 with pure ethanol directly upon collection. Samples for chemical analysis and for 16S rRNA gene amplicon sequencing were taken in triplicates. The collected samples were stored at − 15 °C to avoid changes in the chemical and microbial composition.

### Chemical and operational data

Operational data for each biogas plant were provided by the respective plant operator, including the operating temperature, reactor volume, hydraulic retention time (HRT), reactor type, substrate type, substrate quantity, years of operation, organic loading rate (OLR), additives, biogas yield, methane content, energy production and usage. An overview of the collected data is shown in Supplementary Table 1.

During the sampling phase, some parameters were directly measured on-site, including pH, conductivity, and the oxidation–reduction potential (ORP). The remaining chemical parameters were measured both at the Technische Universität Dresden and at Bioclear Earth B.V. These parameters included total solids (TS), organic total solids (oTS), chemical oxygen demand (COD), ammonium, total Kjeldahl nitrogen (TKN), FOS/TAC, the individual and total organic acids, heavy metals and trace elements. All these parameters were determined using the appropriate norm [21] and the raw data for the chemical parameters are shown in Supplementary Table 2. The methods used to analyse the parameters and the corresponding instruments and units are shown in Supplementary Table 3.

### DNA extraction and high-throughput sequencing

An aliquot of 3 ml of each sample, conserved in ethanol, was centrifuged and washed with sterile phosphate buffered saline (PBS) at least 3 times or until the supernatant was clear. DNA was extracted from the resulting pellets using the NucleoMag DNA Microbiome Kit (Macherey–Nagel, Allentown, PA, USA) with the aid of the AutoPure96 robot for the purification step, following manufacturer's instructions. For cell lysis, the washed pellets were transferred to MN type A bead tubes, together with 700 µL of Lysis Buffer M1. The samples were incubated for 5 min at 70 °C and subsequently shaken for 10 min using a horizontal vortex. DNA was quantified using the Qubit 1 × dsDNA (Thermo Fisher Scientific, Waltham, Massachusetts, USA), and samples were sequenced by Novogene (Cambridge, UK). The extracted metagenomic DNA was used to amplify the hypervariable region V3–V4 of the 16S ribosomal RNA gene. The conserved regions V3 and V4 (470 bp) of the 16S rRNA gene were amplified using the following PCR cycle: initial denaturation at 95 °C for 3 min; 25 cycles of amplification (30 s at 95 °C, 30 s at 55 °C, 30 s at 72 °C); and 5 min of extension at 72 °C [22]. The following primers were used: 341F (5′ CCTAYGGGRBGCASCAG 3′) and 806R (5’ GGACTACNNGGGTATCTAAT 3’). The amplification was carried out using the KAPA HiFi HotStart ReadyMix PCR kit (KK2602). The 16S rRNA amplicons were mixed with Illumina sequencing barcoded adaptors (Nextera XT index kit v2, FC-131-2001), and libraries were normalized and merged. The pools with indexed amplicons were loaded onto the MiSeq reagent cartridge v3 (MS-102-3003) and spiked with 10% PhiX control to improve the sequencing quality. Sequencing was conducted using paired-end 2 × 250pb or 2 × 300pb cycle runs on an Illumina MiSeq device.

### Metataxonomic and statistical analysis

The raw Illumina sequences were loaded into Qiime2 (v. 2021.2.0) [23]. The quality of the sequences was checked using the plugin Demux and the Qiime2-integrated DADA2 pipeline was used for trimming and joining the sequences, removing chimeras and detecting amplicon sequence variants (ASVs) (> 99.9% of similarity). The taxonomy of each sequence variant was determined with the classify-Sklearn module from the feature-classifier plugin, employing SILVA (v. 138) [24] as reference databases for taxonomic assignment. Microbiome data were analysed with the phyloseq package (v 3.16) in R (v 4.2.3) [25]. The abundance of each taxon was correlated to the abundance of the rest of the microorganisms. Moreover, correlations between the microbial community and metadata were calculated. Due to the high number of taxa detected, the statistical tests were performed over the 250 most abundant microorganisms and data were agglomerated to genus level. Spearman’s rank correlation was used to study quantitative variables (content of nitrogen, concentration of acetic acid, etc.) and significant correlations were plotted into heatmaps using the Pheatmap package (v 1.0.12). For these analyses, a normalization of the data using the TSS (total-sum scaling) method was carried out. Qualitative variables (mesophilic, thermophilic) were analysed using the differential abundance test DESeq2 (v 3.16) [26]. Core microbiomes were calculated with the “coremicrobiome” function from the microbiome package [27] in R (v 4.2.3), which provides a list of the most abundant taxa at certain levels of prevalence and abundance, in this case set at 99% prevalence and 0.001, 0.01 and 0.1% abundance. Raw sequences were deposited in the NCBI (BioProject ID:PRJNA1020035, Supplementary Table 5).

## Results and discussion

### Operational process parameters of the AD systems

80 full-scale anaerobic digesters and related systems were investigated based on chemical analyses and DNA sequencing as described in the material and methods. Moreover, the operational parameters of the individual systems were determined. Samples were collected at 45 sites in the Netherlands, Austria and Germany. Sampling points were evenly distributed in the Netherlands. In Germany, the majority of the samples were collected in Eastern Germany, Bavaria and North Rhine-Westphalia. One sample was located in Austria, close to the German border (Fig. 1A).

The AD systems analysed varied in terms of reactor type and general configuration. As shown in Fig. 1B, over 50% of all samples were taken from continuously stirred tank reactors (CSTRs). This high number of CSTR samples is explained by the fact that it is the most common reactor type in the biogas sector [28]. Although most plants are CSTRs, they differ in terms of reactor configurations, size, geometry, and agitation. Some used agitators, some used sprinklers, and some even used a floating roof, which, according to [4], has the potential to stabilize the underlying microbiome. 13% of the samples were taken from plug flow reactors (PFRs). The PFR is designed to allow the feed to flow predominantly axial with minimal radial mixing, allowing for optimum residence time and contact between the feed and the microorganisms [29]. 10% of the samples were from industrial wastewater treatment plants, most of which used an upflow anaerobic sludge blanket reactor (UASB). 6% of the samples were taken from two-staged AD systems, in which the hydrolysis and the acidogenesis phases run separately from the acetogenesis and methanogenesis phases to meet the different requirements of the microorganisms [30]. In order to cover a wider range of AD systems, some rarely occurring digester systems were sampled as well. These include leach bed systems, a unique positive displacement principle system and samples from other parts of the reactor system such as the secondary fermenter and feedstock. The samples used in this study are representative of the most common reactor systems in the biogas sector [29].

The AD systems analysed differed not only in terms of reactor systems, but also in terms of the collected operational process parameters (Supplementary Tables 1 & 2). Figure 1C shows four important operational parameters including temperature, hydraulic retention time, reactor volume and main substrate. The process temperature of the samples taken was in a wide range between 25 and 65 °C, but the majority of the samples were between 35 and 50 °C, at the transition from mesophilic to thermophilic temperature. This range is advantageous for the process and is in line with other studies on the temperature of biogas plants [14]. Higher process temperatures lead to increased microbial activity and thus more biomass is converted to biogas per unit of time, which is why temperatures above 35 °C are beneficial [31]. However, increased degradation rates that result from higher temperatures can lead to accelerated release of organic acids and other potentially process-inhibiting metabolites [32]. The HRTs ranged from one day for the UASB and the first stage of the two-stage AD systems to 170 days for the conventional CSTRs and secondary digesters. Although the range of HRTs is relatively broad, most reactors have an HRT between 15 and 60 days. Interestingly, small reactors tended to have low HRT, while substrates also had an impact on HRTs. In this regard, Fig. 1 C shows that organic waste, sewage sludge, and industrial wastewater had short HRTs of about 20 days, which is consistent with the literature and suggests that these substrates can be converted quickly [33]. This is followed by a range between 20 and 80 days for energy crops, which tend to have higher fibre contents and therefore take longer to degrade [34]. The last group are the residual crops including grass silage, wheat straw and green waste, which required HRTs of 80–180 days due to the high proportion of lignin and fibre components that are difficult to degrade [35]. The use of manure and/or sewage sludge as a part of the substrate was found in all digesters as it is a well-suited seed sludge, which is already enriched in some key organisms for the methanogenic biogas process [36]. The AD systems investigated in this study show a high diversity in terms of substrates used, reactor systems and operational parameters. In addition to the main substrates mentioned above, rare cases with substrates such as slaughterhouse waste, grease, industrial wastewater, supermarket waste, and unique residual materials such as sudan grass, sugar beet, millet and manures from goats and horses were used (Supplementary Table 1). Furthermore, the normalized production of biogas and energy are important parameters for evaluating the efficiency of biogas plants. Unfortunately, this data could not be obtained from all plant operators, which is why it was not possible to obtain clear results due to its incompleteness. All varying parameters mentioned, such as reactor systems and configurations, temperature, retention time, volume, and feedstock underline the high diversity of the presented set of samples. Samples from this study represent the majority of industrially applied plant types in the biogas sector. The combination with the operational, chemical and taxonomic data provides one of the most comprehensive and diverse data sets on biogas plants to date (Supplementary Tables 1 and 2).

### Chemical parameters of the AD systems

**Fig. 2:**
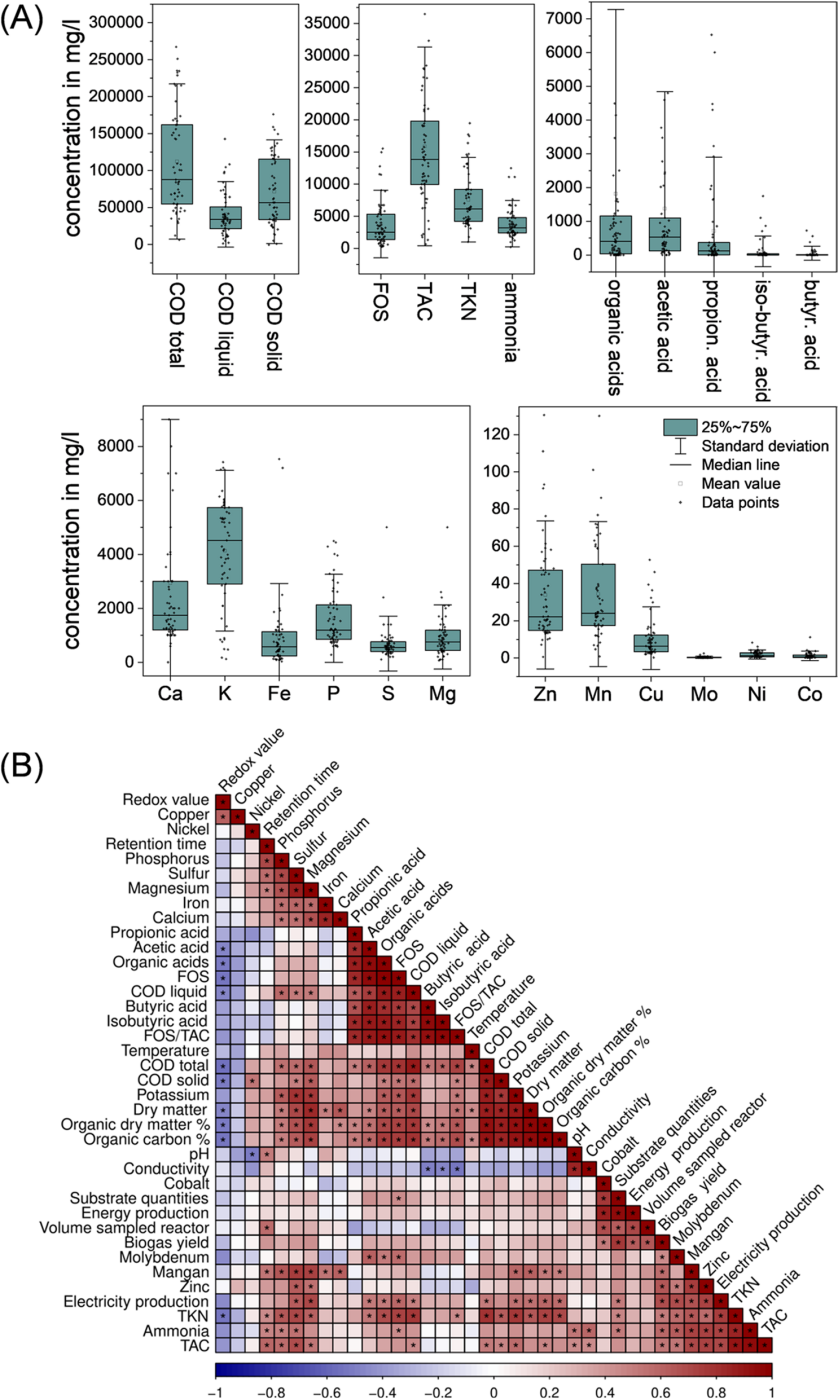
**A** Boxplots depicting the concentration of the main chemical parameters. Box size indicates the distribution of values between 25 and 75% of the values. **B** Spearman correlation analysis comparing the chemical variables. Positive correlations (i.e. positive Spearman's rank correlation coefficients) are highlighted in red, negative correlations are highlighted in blue and the black dots indicate whether the respective correlation is significant

A comprehensive set of chemical parameters was collected for all the samples analysed in this study (Supplementary Tables 2, 3). Due to the wide variety of operational parameters, high variations of the measured chemical parameters were observed. Total volatile fatty acids (TVFAs) exhibited a large upward scatter, which could be explained due to the different systems, their specific configurations, varying feeding conditions and individual conditions in respect to the physical–chemical parameters (Fig. 2A). The strong variations of key parameters such as VFAs among reactors indicate that the sampled reactors bear a high diversity of microbial habitats. Generally, one-stage systems are operated and designed to keep VFAs as low as possible. In contrast, the first stages of two-stage AD reactors have high concentrations of VFAs. In such systems, it is aimed to separate hydrolysis/acidogenesis from acetogenesis/methanogenesis to optimize the respective process conditions. To suppress hydrolysis/acidogenesis, the HRT is minimized. As a result, VFA concentrations increase and the pH drops, which impairs methanogenic archaea and related acetogens. This concept has been investigated already for 50 years [30] and Holl et al. mention that 50 years of research have not led to industrial solutions. This is not quite correct, since three industrial two-stage AD systems were investigated in the present study. However, chemical parameters (Supplementary Table 2) revealed the presence of methanogenesis in the first stage despite high OLRs. To the surprise of the respective plant operators, the pH was in all cases above 7.0 in all hydrolysis/acidogenesis stages, which is beneficial for methanogenesis. Methane losses in a separated acidification stage have also been described by other scientists [37]. One of the plant operators highlighted that they had noticed high ratios of methane in the hydrolysis/acidification stage, which is not expected. The operator tried to prevent this by increasing the OLR further, however, with a maximal OLR of 44.6 kg/m^−3^/d^−1^, there were still methanogens which indicates, that they were able to adapt due to adaptive evolution. A further increase in the OLR was not possible without overloading the pumps of the respective plants. Thus, the statement by Holl et al. could be reformulated in such a way that although several two-stage industrial-scale plants have already been built, no industrial solutions are known that follow the concept of a two-stage biogas plant in terms of process technology. Apart from two-stage AD systems, high VFA concentrations were also observed in PFR. Although these reactors are not considered classical two-stage systems, they still have high levels of VFAs due to the high OLR. For this reason, all PFR sampled in this study have a downstream methane stage to increase the degradation efficiency.

Despite the large upward scatter for VFAs, most digesters had a rather low concentration of VFAs. Therefore, it can be assumed that there is no significant inhibition of the methanogenic activity due to VFAs. The average acetate concentration was 1373 mg/l, which is far below inhibiting conditions of ~ 2400 mg/l for one-stage systems [38]. Despite maximum OLR and high VFA concentrations in two-stage systems, long-term inhibition of methanogenesis was not observed there. The threshold mentioned by Franke-Whittle et al. is therefore dependent on the type of plant and the degree of adaptation of methanogenic archaea. The average butyrate concentration was 52 mg/l, well below inhibiting conditions, which start at 1800 mg/l. Propionic acid was the VFA that was closest to an inhibiting range, with an average concentration of 716 mg/l (inhibition starts at 900 mg/l) [38, 39]. All other parameters showed lower deviations and thus a lower dispersion of the distributions of the 25–75% quantile but with a tendency towards higher values. It must be emphasized that the values at which inhibition occurs vary depending on many factors, for example, due to microbial adaptations and the degree of protonation of the VFA salts.

A wide dispersion can be also observed for the total COD, the COD from the solid’s fractions, as well as for the total inorganic carbon, Ca, K and P. According to the literature, these parameters are strongly influenced by the OLR, which means mainly by the substrate, the substrate quantities and the hydraulic retention time [40]. In contrast, the quantile for COD liquid, volatile fatty acids determined by two-point titration (FOS), total nitrogen according to Kjeldahl (TKN), ammonia and all other trace elements is narrow. Strong deviations from the norm of these parameter values are often accompanied by process interruptions. Therefore, these parameters are intended to be kept constantly low [41]. Furthermore, most of them are limited by physicochemical effects such as solubility limits, precipitation and pH dependence. In addition to the parameters shown in Fig. 2, the following additional parameters were determined and presented as the mean with a standard deviation of all samples: pH = 7.6 ± 0.4; ORP = − 374 mV ± 77; conductivity = 16.6 µS/cm ± 8.5; total solids (TS) = 10.1% ± 6.7; and total organic solids (oTS) = 6.5% ± 3 (Supplementary Table 2). Compared to the literature, it is noticeable that the values for total COD and COD solids as well as TS and oTS are comparatively high and more variable [42] which can be explained by the predominant utilization of plant-based materials like energy crops and residual crops in agricultural biogas plants [43]. The correlation between high TS and high COD has been demonstrated using a Spearman correlation analysis (Fig. 2B) and is consistent with the literature [44]. It is striking that there are predominantly significant positive correlations among most of the parameters (Fig. 2B), which can be explained by the cascade nature of the process in which different processes build on each other [45]. These positive correlations include dependencies between the individual VFAs, which can be explained by the fact that VFAs are degraded stepwise from medium-length fatty acids to short-chain organic acids [46, 47]. In addition, acids correlate positively with COD, TS, oTS and TKN. All of these parameters are indicators of nutrient availability, so it is reasonable to assume that an increase in nutrient availability will also lead to an increase in hydrolytic activity and the formation of more VFAs. All values that directly or indirectly indicate the amount of organic matter, such as COD, TS, and oTS, correlate positively with TKN and the trace elements P, S, Mg and K. Both trace substances and nitrogen naturally occur in the substrate, especially in manure and plant components, thus explaining this correlation. In particular, high levels of these elements can be detected in the solid components. The trace elements Ni, Mn and Mo also showed significant positive correlations with each other. As many plant operators use additives containing these trace elements, this correlation can be justified by that. Negative correlations between chemical variables mainly concerned the site parameters, i.e. pH, conductivity and ORP, with only the ORP showing significant negative correlations. Overall, the chemical data confirm that the samples constitute a very heterogeneous data set. In particular, parameters such as organic acids, COD and some trace elements showed a wide range of possible values, displaying many significant positive correlations according to the Spearman correlation analysis, but without exceeding the inhibition values indicated in the literature.

### Taxonomic profiling of the AD systems

**Fig. 3:**
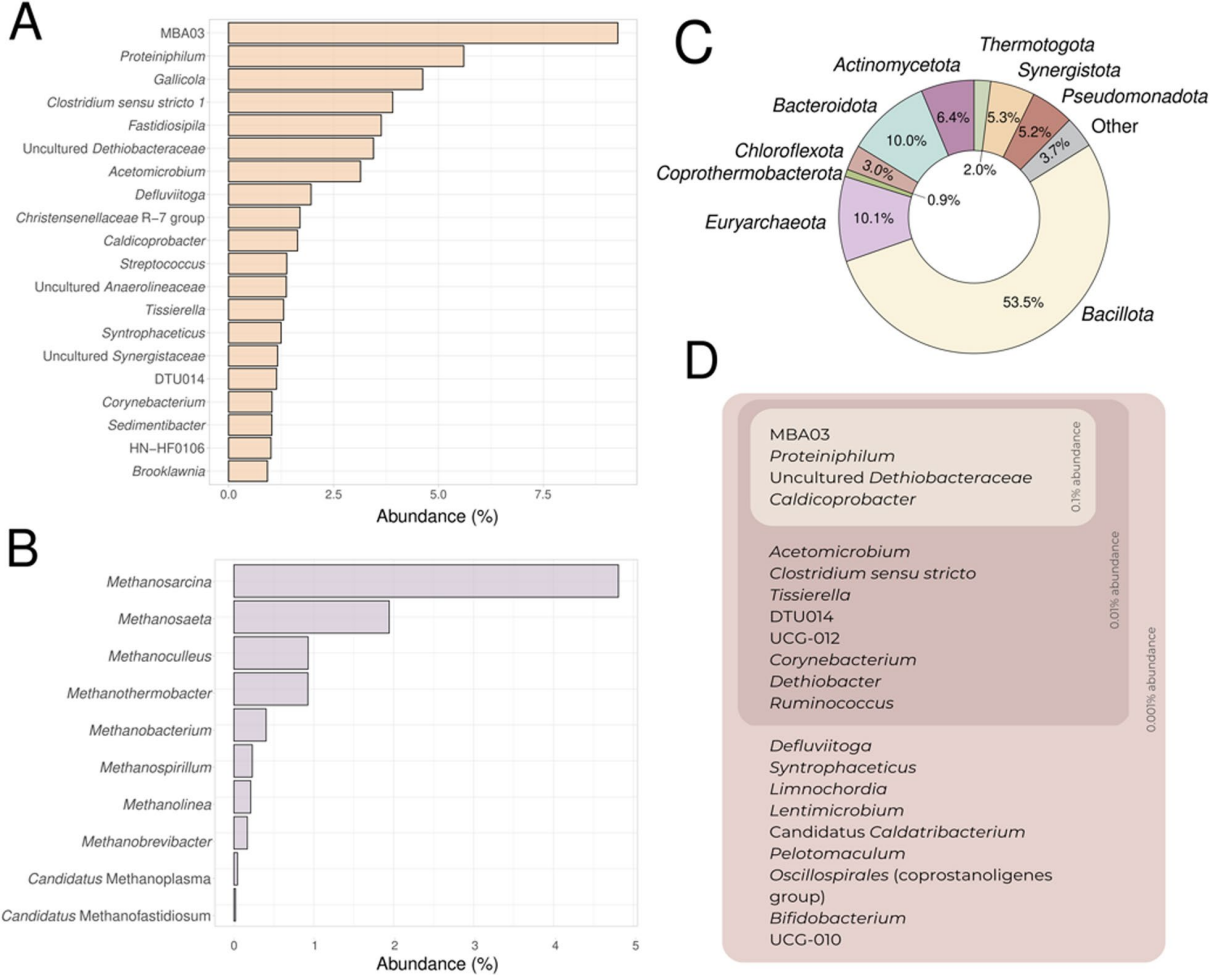
Illustration of the highest relative abundance for all samples on average at genus level for bacteria (**A**), genus level for archaea (**B**) and phylum level for bacteria and archaea (**C**). Visualisation of the core micobiome present in all samples with a minimum abundance of 0.1 %, 0.01 % and 0.001 % (**D**)

A total of 42,939 different amplicon sequence variants (ASVs) were detected in the entire dataset, accounting for 1858 genera and 61 phyla. The average richness and Shannon indices at the ASV level were 1019.13 ± 279.65 and 4.43 ± 0.76, respectively. At the phylum level, the average AD reactor was dominated by *Bacillota* (53.5%) followed by *Bacteroidota* (10.0%) and the archaeal phylum *Euryarchaeota* (10.1%). *Actinomycetota* (6.4%), *Pseudomonadota* (previously *Proteobacteria*) (5.2%), *Synergistota* (5.3%) and *Chloroflexi* (3.0%) were also detected in lower abundances (Fig. 3C). These results are consistent with previous findings reporting that *Bacteroidota* and *Bacillota*, especially *Clostridia* and *Bacilli* classes dominate AD processes [48, 49]. *Pseudomonadota* and *Synergistetes* phyla are usually found in lower abundances but are still prevalent [48], as well as *Acidobacteria*, *Actinomycetota* and *Chloroflexi*. In our samples, four phyla covered 80% of the average population and six phyla covered 90% of the average population.

At the genus level, taxonomic profiles were dominated by MBA03 (9.3%), followed by *Proteiniphilum* (5.6%), *Gallicola* (4.6%) and *Clostridium* sensu stricto (3.9%) (Fig. 3A). Other abundant genera were *Acetomicrobium*, an uncultured *Dethiobacteraceae*, *Syntrophaceticus*, DTU014 and *Caldicoprobacter*, which were also present in the core microbiome (Fig. 3D, Supplementary Table 4A). *Archaea* represented nearly 10% of the total microorganisms in the samples, with a maximum abundance of 48% in one sample. *Methanosarcina* was on average the most abundant archaeal genera (4.8%) (Fig. 3B). This genus was detected in 78/80 samples, indicating a high prevalence in full-scale reactors. Other abundant archaea were *Methanothrix* (1.9%), *Methanoculleus* (0.9%) and *Methanothermobacter* (0.9%) (Supplementary Table 4A). It is important to highlight that some archaeal genera were assigned to phylum *Halobacterota* according to the SILVA database (v. 138) [24], while the “List of Prokaryotic names with Standing in Nomenclature” (LPSN) [50] classifies them as members of the phylum *Euryarchaeota*. In these cases, the taxonomy was manually corrected to match de LPSN criteria.

Previous studies have identified members of the orders *Methanosarcinales*, *Methanobacteriales* and *Methanomicrobiales* as the dominating methanogenic archaea in AD systems [48, 51] Moreover, *Methanosarcinales* occurs in the core microbiome and can be detected in averages up to 5% [52]. According to the results, the MBA03 genera and *Caldicoprobacter* from the *Bacillota* phylum, the genus *Proteiniphilum* from the *Bacteroidota* phylum, and an uncultured organism from *Dethiobacteraceae* family compose the core microbiome of anaerobic digesters, as they are present in 100% of the processed samples with an abundance higher than 0.1% (Fig. 3D). When looking at the functions of the detected core bacteria in AD, two main roles can be identified. On one hand, the microorganisms of the MBA03 genera, the DTU14 genera and a genus of the *Dethiobacteraceae* family show high ammonia tolerance and syntrophic acetate oxidation (SAO) activities, thus contributing to hydrogenotrophic methanogenesis [8]. On the other hand, MBA03, *Caldicoprobacter*, *Proteiniphilum*, *Acetomicrobium* and *Defluviitoga* show mainly hydrolytic activities [8, 53]. MBA03 and *Defluviitoga* can degrade complex carbohydrates such as xylan, cellulose and lignocellulose [54, 55]. *Proteiniphilum* and *Acetomicrobium* can degrade both peptides and complex carbohydrates [8, 56, 57], and *Caldicoprobacter* is considered to have the ability to hydrolyse lipids, peptides and carbohydrates [58]. It must be highlighted that MBA03 is the only taxon that performs both major functions in the system, displaying both syntrophic acetate oxidation (SAO) and cellulolytic and xylanolytic activities, potentially playing a key role in AD systems. [8, 54, 59].

### Multivariate Spearman correlation analysis

**Fig. 4:**
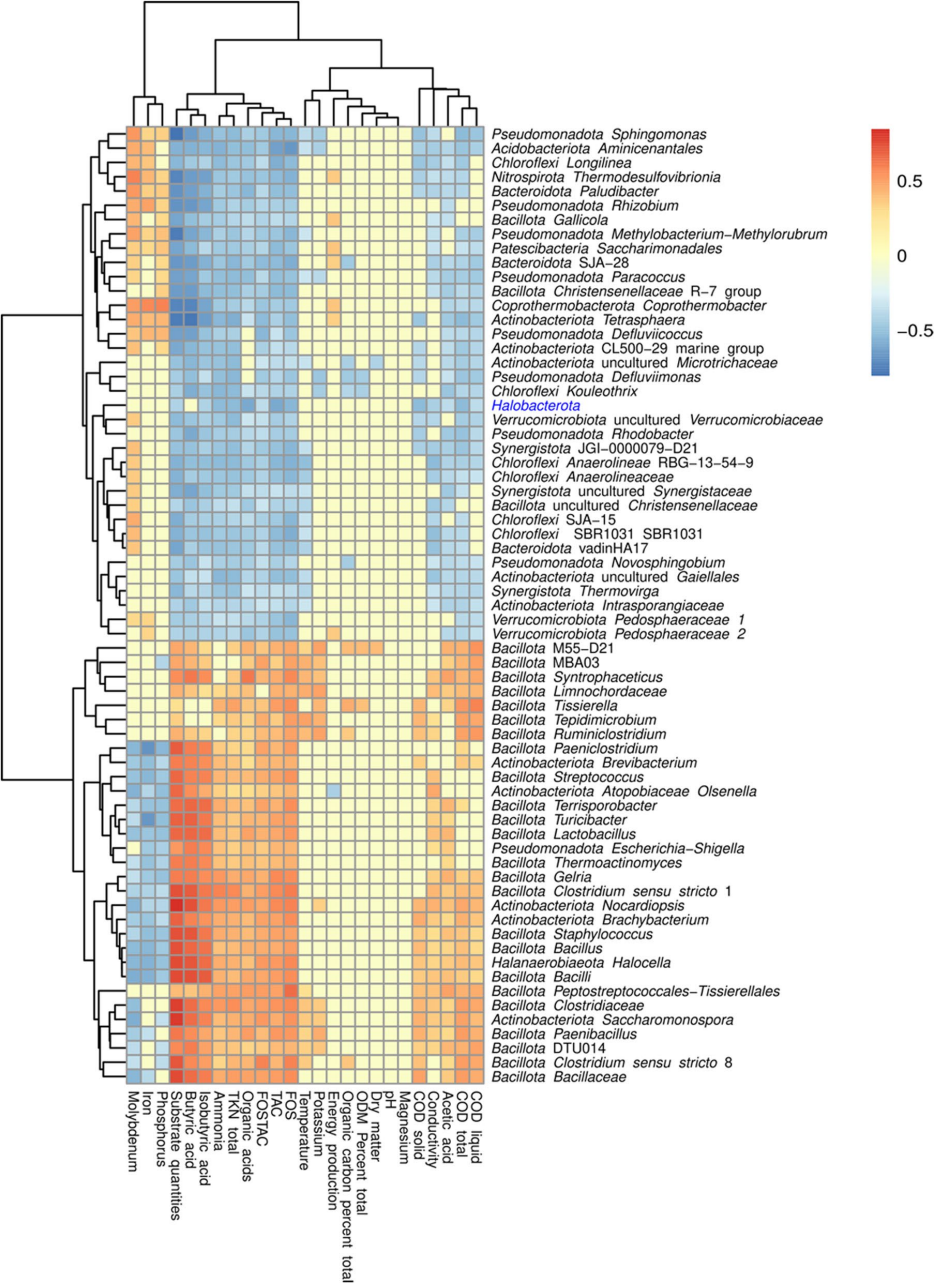
Spearman correlation analysis of 66 genera showing at least 14 correlations with chemical variables were plotted in a heatmap using the complete linkage method for hierarchical clustering. In this heatmap, red colours represent positive correlations and blue colours represent negative Spearman correlations. The full description of all the correlations detected is shown in Supplementary Table 4B. All the correlations plotted are significant (p-value < 0.05). *Halobacterota* is highlighted because the Silva database incorrectly assigns the methanogen *Methanoculleus* to the genus

In this study, Spearman correlation analysis showed many associations between microorganisms and the analysed chemical variables, whereas for the operational parameters, only temperature and substrate amounts showed significant correlations with the taxonomic profiles (Fig. 4, Supplementary Table 4B).

Hierarchical clustering revealed two distinct clusters of microorganisms showing similar trends concerning chemical variables. The first cluster showed a higher number of bacteria belonging to phyla *Pseudomonadota*, *Actinomycetota*, *Chloroflexi* and *Bacteroidota*, reporting negative correlations with all the organic acids, nitrogen and COD. These bacterial genera are involved in hydrolysis by degrading complex molecules such as chitin, peptide, lignocellulose and/or cellulose [8, 60]. When acidogenesis begins after the hydrolytic reactions, the concentration of organic acid begins to increase, while the concentration of complex macromolecules decreases. Consequently, the abundance of these bacteria may decrease accordingly. Both phyla, *Chloroflexi *and *Bacteroidota* are known to be typical acidogenic classes with hydrolytic activity, capable of metabolizing simple compounds such as amino acids, glycerol, glucose and complex polysaccharides [8, 61, 62]. Within *Bacteroidota*, a higher presence of the *Paludibacteraceae* family (i.e. *Paludibacter*) has been reported in the earlier steps of AD processes [8]. Members of the *Anaerolineaceae* family (i.e. *Longilinea*, *Anaerolinea*), which is the most abundant in the *Chloroflexales* order, metabolize polysaccharides such as pectin and xylan and produce acetic and lactic acid and hydrogen [63]. Some microorganisms belonging to the Bacillota phylum*,* such as *Gallicola,* were also found in the first cluster, negatively correlating with organic acids. These non-saccharolytic genera can metabolize peptone and amino acids to organic acids [8, 64, 65]. There may be two reasons for this negative correlation: first, *Gallicola* can occur in a small window of organic acid concentration, and with increased organic acid content, the activity decreases. Second, *Gallicola* only metabolizes peptides, so its occurrence is limited by the presence of peptides [64].

The second cluster of microorganisms showed positive correlations with organic acids (i.e. acetic acid, butyric acid and iso-butyric acid), ammonia, nitrogen, COD, substrate quantities and negative correlations with Fe, P and Mo. Despite being close to the inhibition point, propionic acid has no significant positive or negative correlation with the shown microorganisms. According to some studies, the *Syntrophaceticus* genera, MBA03, DTU014 and the *Dethiobacteraceae* family within the Bacillota phylum are potential syntrophic acetate oxidizing bacteria (SAOB) [66–68]. Therefore, these bacteria would oxidize acetate to CO_2_ and H_2_, which would then be used to produce methane by hydrogenotrophic archaea. In addition, the orders DTU014 and MBA03 and members of the *Dethiobacteraceae* family have been reported to increase their abundance with the progression of AD [8], which fits with the hypothesis that the genera in the first cluster represent microorganisms performing hydrolysis and acidogenesis. In contrast, the microorganisms in the second cluster are involved in the steps closer to acetogenesis and methanogenesis.

**Fig. 5:**
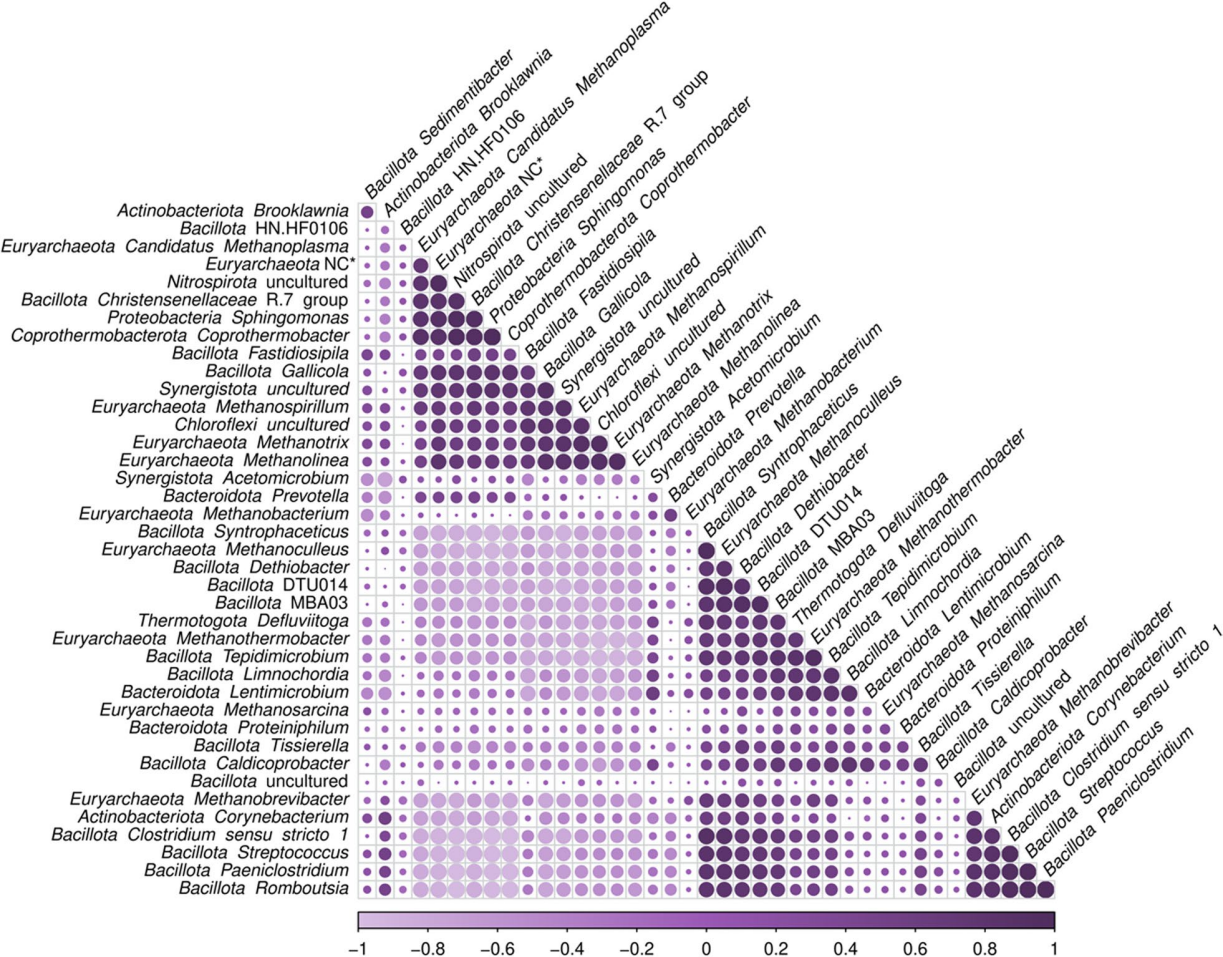
Correlations between the most abundant 30 bacteria and 10 archaea. The different shades of purple show the value of the correlation (legend below), while the size of the dot shows the significance of the correlation. The full description of all the correlations detected is shown in Supplementary Table 4C

Spearman correlation analysis with taxonomic data showed which prokaryotes were influenced by the abundance of other microorganisms, providing relevant information about the relationship between taxa during the AD processes (Fig. 5, Supplementary Table 4C). The *Methanothermobacter* genera, *Methanoculleus* genera and *Methanosarcina* genera*,* which are hydrogenotrophic methanogens [48], positively correlated with potential SAOB, such as DTU014, MBA03, *Syntrophaceticus* and *Dethiobacter*, forming a cluster (Fig. 5), whereas *Methanothrix,* an acetoclastic methanogen, negatively correlated with these bacterial genera. This supports the hypothesis that the abundance of SAOB is tightly related to hydrogenotrophic archaea; these bacteria would compete for acetate with acetoclastic methanogens and use acetate to generate hydrogen, thus producing a shift of the balance towards hydrogenotrophic methanogenesis [48, 69]. The above explanation makes sense if you consider *Methanothrix* as an acetoclastic methane producer. However, it is important to emphasize that a switch from acetoclastic metabolism to hydrogenotrophic metabolism has recently been described for *Methanothrix* [70]. The relationships described above could therefore be more complex.

Most *Chloroflexota* genera, mainly dominated by the *Anaerolineaceae* family, negatively correlated with the genera in the cluster of syntrophs (DTU014, MBA03, *Syntrophaceticus*, *Clostridium *sensu stricto 1), while displaying positive correlations with *Christensenellaceae*-R7-group and *Gallicola*. Some bacteria within the syntrophic cluster were also positively correlated with temperature (Fig. 4), indicating that their growth is favoured in thermophilic conditions. In this sense, members of class *Clostridia* are known to be more abundant in high temperatures and increasing ammonia levels [71]. Although high levels of ammonia can cause the inhibition of the process, SAOB seemed to be particularly tolerant to ammonia (Fig. 4), and this may result in a shift to hydrogenotrophic methanogenesis since these methanogens grow in syntrophy with SAOBs [48]. Interestingly, other authors have also reported positive correlations between ammonia and hydrogenotrophic methanogens and SAOB [69, 71].

### Influence of temperature and nitrogen content on the microbiome

Both temperature and ammonia variables were used as categorical variables for further comparisons. As reported by several authors, a reactor was considered “mesophilic” when displaying operational temperatures up to 45 ºC, and “thermophilic” if the temperature was above 45 ºC [48, 72].

In total, 41 genera were significantly more abundant in thermophilic conditions, while 171 increased their abundance in mesophilic conditions (Supplementary Table 4D). Different archaea, such as *Methanomethylovorans*, *Methanothrix* (previously *Methanosaeta*), *Methanolinea* and *Methanospirillum,* were more abundant in mesophilic conditions. The methanogenic community is especially sensitive to process instability [48, 73], and thermophilic reactors have been reported previously to be less stable in comparison to mesophilic ones [74]. Moreover, the *Fastidiosipila* genera and the *Petrimonas* genera were more abundant in mesophilic conditions, which is consistent with previous reports [53]. According to the results obtained (Fig. 4), the most abundant microorganisms in thermophilic conditions belong to the *Bacillota* phylum (70% of them to class *Clostridia*). Specifically, thermophilic reactors were enriched in MBA03, DTU014, *Syntrophaceticus*, *Lentimicrobium*, *Defluviitoga* and *Tepidimicrobium* (Supplementary Fig. 1).

It is well known that high amounts of nitrogen, especially ammonia, can inhibit the AD process [75, 76]. However, there is no consensus on the concentration above which this molecule negatively affects biogas production, as the ammonia concentration depends on the pH value, the temperature and the processing state of the ammonia, which in turn depends on the substrate and the underlying microbiome. Some authors have reported that a critical threshold concentration of ammonia, which causes first inhibition and subsequently toxicity is between 1500 and 7000 mg/l [75]. To prove the effect of ammonia levels on the microbial community, the quantitative variable was divided into “low ammonia content” for values up to 5000 mg/l and “high ammonia content” for values above 5000 mg/l and the differential abundance in both situations was calculated (Supplementary Table 4E). In low ammonia conditions, a higher abundance of archaeal genera (such as *Methanosarcina*, *Methanothrix*, *Methanospirillum*, *Methanoplasma* or *Methanolinea*) was detected. There is previous evidence that high ammonia conditions can affect the whole microbial community, but methanogenic archaea are the ones which suffer this stress the most, particularly acetoclastic methanogens [69, 77]. Regarding the bacterial community, *Clostridium *sensu stricto 1, *Proteiniphilum* and *Defluviitoga*, among others, were more abundant in reactors with ammonia concentrations above 5000 mg/l (Supplementary Fig. 2). Both *Clostridium *sensu stricto 1 and *Proteiniphilum* have been reported to be abundant in high ammonia conditions [77, 78]. This could be due to their main ability to degrade proteins/amino acids [8]. In addition, *Acetomicrobium*, *Christensenellaceae* R7 group and *Gallicola* were more abundant in lower ammonia conditions (Supplementary Fig. 2).

### Relation between microbial, operational and site-related factors

To study the relationship between the microbial communities and the substrates used in AD, the feedstocks were divided into three different groups, based on their chemical composition [32]: organic biological waste (cluster “biowaste”), energy crops and agricultural animal waste (cluster “agricultural waste”) and wastewater sludge and industrial wastes (cluster “industrial waste”). It is important to highlight that the feedstocks used in full-scale reactors are a mixture of substrates from different sources, which complicated the analysis. However, some relevant conclusions were obtained. The *Pseudomonadota* phylum was more abundant in the AD systems inoculated with substrates coming from industrial waste, this phylum has been previously related to UASB reactors treating sludges [48]. Moreover, some core genera such as MBA03 and UCG-010, together with the hydrogenotrophic archaea *Methanoculleus* showed higher abundances in agricultural wastes and biowaste-based feedstock. In contrast, *Syntrophaceticus*, *Clostridium *sensu stricto 1 and the hydrogenotrophic archaea *Methanothermobacter* were more abundant in substrates from agricultural origins (Supplementary Fig. 4) which was previously reported in the literature [79].

The operational design of the AD process is another factor affecting the resulting microbial community and the final biogas yield. Different parameters, such as shear, granule formation, hydraulic retention time, liquid upflow velocity or feed rate [80] can be determined for one microbial community to find their optimal conditions for growth. Regarding reactor type, the most common ones among the samples were continuously stirred tank reactors (CSTR; 44 samples), plug flow digesters (PFD, 10 samples), upflow anaerobic sludge blanket digesters (UASB; 8 samples) and two-stage AD reactors with hydrolysis (5 samples). The differential expression analysis revealed that *Syntrophaceticus* and *Caldicoprobacter* were overexpressed in CSTRs, *Coprothermobacter* was overexpressed in PFRs, while other microorganisms present in the core microbiome, such as *Fastidiosipila*, *Gallicola* and *Christensenellaceae* R7 group were more abundant in UASB digesters (Supplementary Fig. 5). Two hydrogenotrophic archaea, *Methanothermobacter* and *Methanolinea*, were overexpressed in CSTRs and two-stage reactors, respectively, while *Methanothrix* was more abundant in UASB reactors. Genera from the *Pseudomonadota* phylum were particularly abundant in UASB, which is in line with the results obtained for substrates coming from industrial wastes and with previous developments [81].

Statistical differences were found in the taxonomic profiles between Dutch and German digesters (Supplementary Fig. 6, Supplementary Table 6). This could have several reasons (i.e. unequal sampling of the different reactor types or differences in the substrates) and should be investigated in the future.

### Limitations and outlook

Despite research efforts made in recent years, AD is still a microbial black box due to the complexity of microbial transformations, and interactions, the variability of process designs, and the high number of operational and chemical variables that affect the underlying microbiomes. For this reason, the present study faced three challenges. First, the substrates used in full-scale reactors were diverse and complex, consisting mainly of mixtures of different feedstocks in unknown proportions, which made it difficult to determine the true influence of each substrate on the AD microbiomes. Moreover, this study focused on studying bacteria and archaea, but viruses, protozoa or anaerobic fungi, are also important members of the AD microbiomes and their role in industrial biogas production should be addressed in future works. Finally, it must be highlighted that not all the industrial plant operators measured and evaluated the relevant operational parameters like methane content, biogas yield, etc., which makes it difficult to compare. This prevented the establishment of correlations between methane production/efficiency and the various chemical, taxonomic and operational parameters.

## Conclusions

The core microbiome of 80 full-scale anaerobic digesters consisted of MBA03, *Proteiniphilum*, a member of *Dethiobacteraceae* and *Caldicoprobacter*. *Methanosarcina* was detected in 98% of the samples. Based on Spearman analyses of the multivariate data sets, different clusters of microorganisms were identified. For the purely taxonomic analysis, two exclusive clusters of microorganisms were identified: one group included microorganisms associated with acetoclastic archaea, while another group was associated with hydrogenotrophic archaea and related to syntrophic acetate-oxidizing bacteria. However, the multivariate analysis based on the taxonomic and chemical parameters revealed two exclusive microbial clusters: one including hydrolytic and acidogenic microorganisms, and the other comprising bacteria related to acetogenesis (i.e. syntrophic acetate-oxidizing bacteria). In particular, the chemical parameters organic acids, ammonia, total nitrogen, chemical oxygen demand, the trace elements Fe, Mo and the macronutrient P significantly influenced the formation of the respective AD microbiome. Among the operational parameters, temperature, reactor type, substrate composition and quantity have a major influence on the microbiome, but only on the formation of certain microorganisms in the AD system. Further, the reactor type with separate acidification stood out due to its unexpected behaviour. Despite maximizing the organic loading rate in the hydrolytic pretreatments, these stages converted into extremely robust methane production units. Microbial clusters were generally highly dynamic depending on whether taxonomic, chemical or operational parameters were combined. Overall, this work identifies the most important microbial players for a wide range of AD systems.

### Supplementary Information

Supplementary file 1: Supplementary Figure 1. Differential expression of eight bacterial genera in different temperature conditions. Blue represents mesophilic condition (<45 ºC) and pink represents thermophilic condition (≥45 ºC). The statistical analysis was performed with DESeq2 in RStudio.

Supplementary file 2: Supplementary Figure 2. Differential expression of six bacterial genera in reactors with different nitrogen content. Yellow represents high ammonia content (>5000 mg/l) and green represents low ammonia content (<5000 mg/l). The statistical analysis was performed with DESeq2 in RStudio.

Supplementary file 3: Supplementary Figure 3. Relative abundance of archaea and bacteria in the processed samples.

Supplementary file 4: Supplementary Figure 4. PCoA analysis of the three substrate groups (A) and different reactor types (B). The low p-value (p-value=0.001) obtained with the PERMANOVA test (Adonis2) shows that there is a significant difference between the groups. Both tests were performed in RStudio.

Supplementary file 5: Supplementary Figure 5. Most abundant genera in each reactor type.

Supplementary file 6: Supplementary Figure 6. Most abundant genera in each country, discovering country-specific microbiomes.

Supplementary file 7: Supplementary Tables 1-3. All metadata.

Supplementary file 8: Supplementary Tables 4A-F. All multivariate analysis.

Supplementary file 9: Supplementary Table 5. Sequence IDs of uploaded bioprojects.

Supplementary file 10: Supplementary Table 6. Country-specific comparison between Germany and the Netherlands. Differential analysis between both groups.

## Data Availability

Raw sequences were deposited in the NCBI (BioProject ID: PRJNA1020035). All other data generated or analysed during the trial are included in this published paper.

## Abbreviations

ASV: Amplicon sequence variants
CSTR: Continuous stirred tank reactor
min: Minutes
OLR: Organic loading rate
OTU: Operational taxonomic unit
PCR: Polymerase chain reaction
PFR: Plug flow reactor
SAOB: Syntrophic acetate oxidizing bacteria
UASB: Upflow anaerobic sludge blanket reactor
TSS: Total-sum scaling
