# Supplementary figures and images for: Multivariate comparison of taxonomic, chemical and operational data from 80 different full-scale anaerobic digester-related systems
Source: Biotechnol Biofuels Bioprod. 2024 Jun 20;17:84. doi: 10.1186/s13068-024-02525-1 (PMC11191226; doi:10.1186/s13068-024-02525-1)

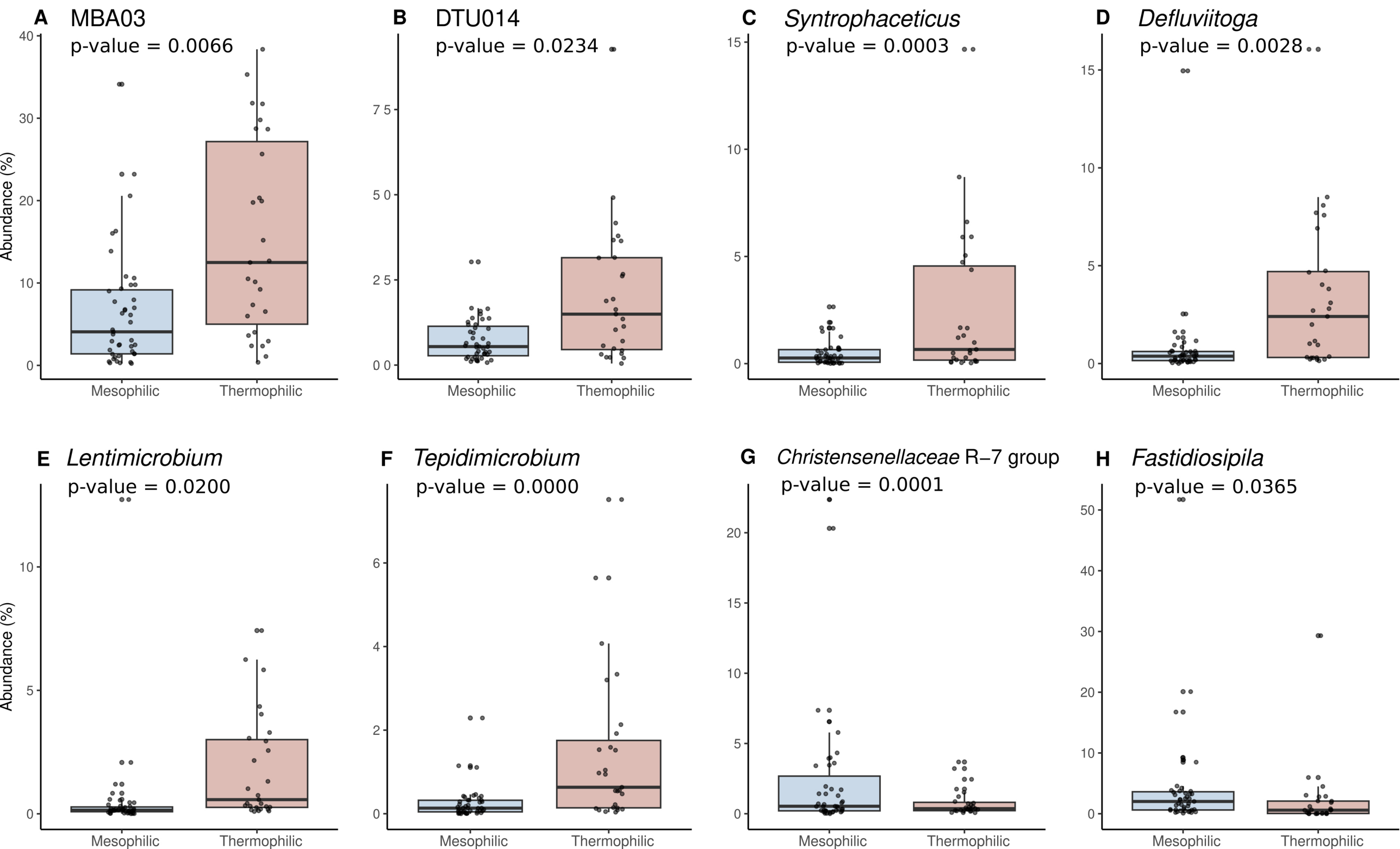

Supplement: Supplementary file 1 — Supplementary Figure 1. Differential expression of eight bacterial genera in different temperature conditions. Blue represents mesophilic condition (<45 ºC) and pink represents thermophilic condition (≥45 ºC). The statistical analysis was performed with DESeq2 in RStudio. [file 13068_2024_2525_MOESM1_ESM.pdf]

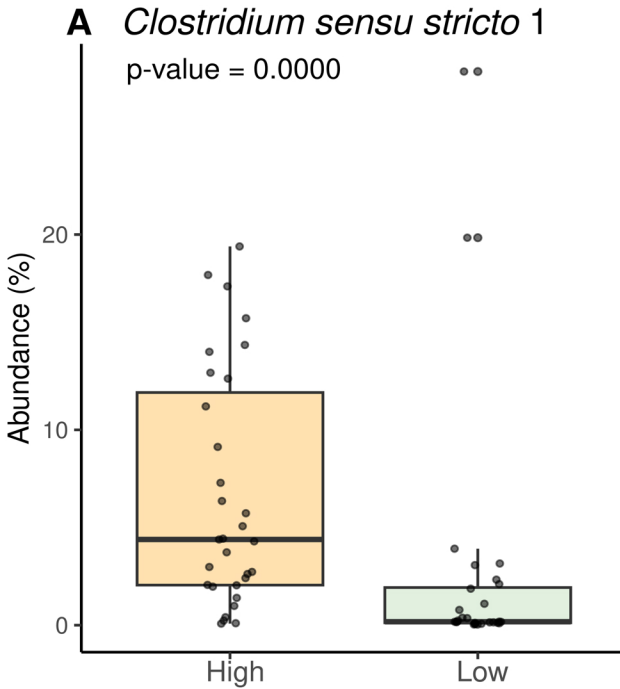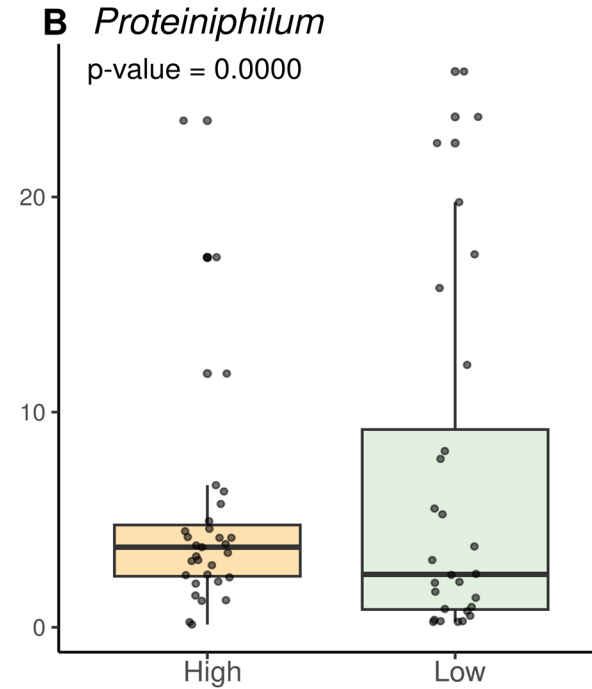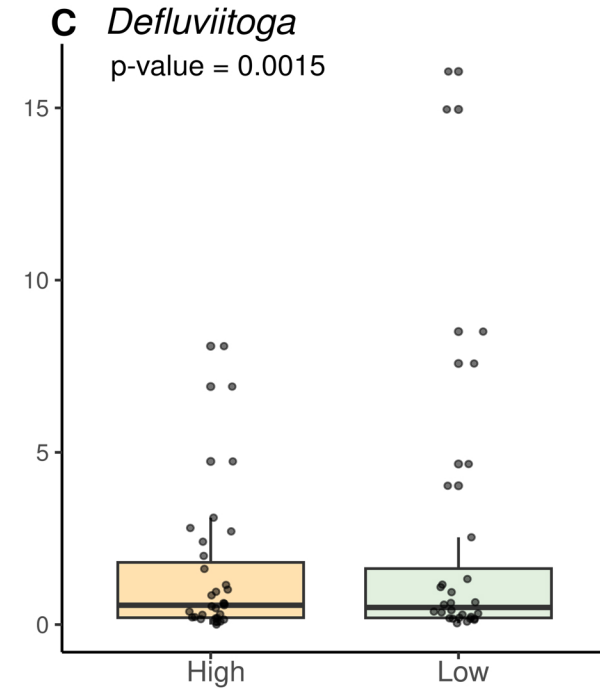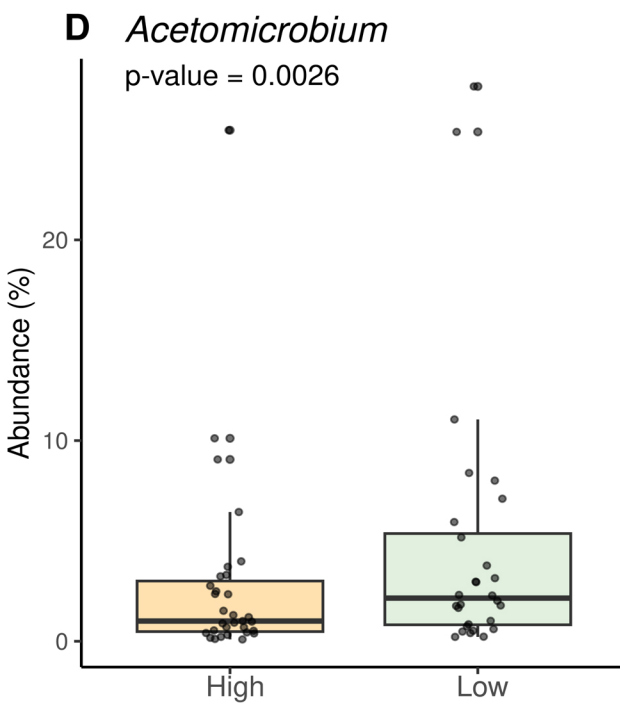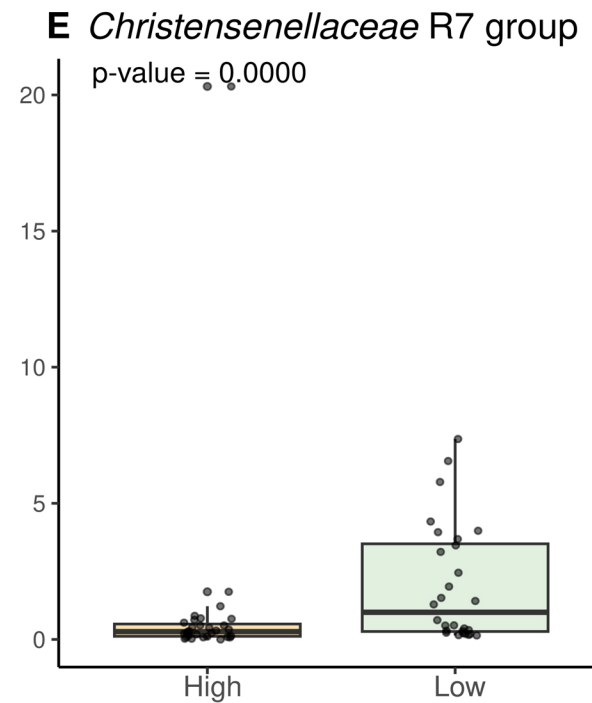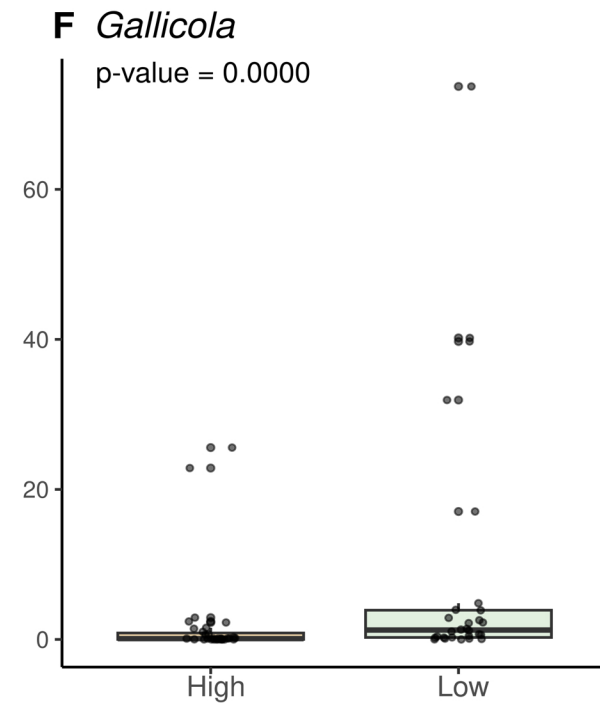

Supplement: Supplementary file 2 — Supplementary Figure 2. Differential expression of six bacterial genera in reactors with different nitrogen content. Yellow represents high ammonia content (>5000 mg/l) and green represents low ammonia content (<5000 mg/l). The statistical analysis was performed with DESeq2 in RStudio. [file 13068_2024_2525_MOESM2_ESM.pdf]

Abundance

100  
75  
50  
25  
0

Archaea

Bacteria

Taxa

Archaea

Bacteria

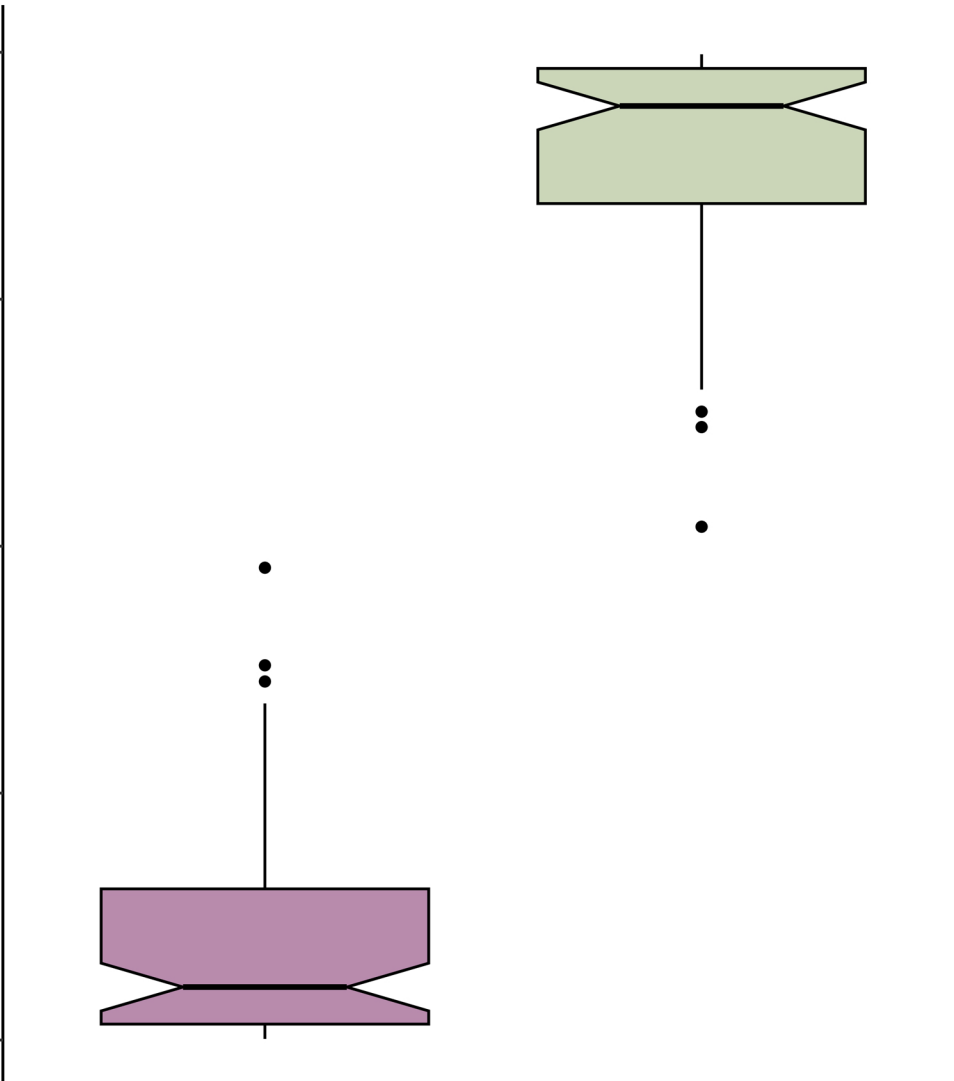

Supplement: Supplementary file 3 — Supplementary Figure 3. Relative abundance of archaea and bacteria in the processed samples. [file 13068_2024_2525_MOESM3_ESM.pdf]

(A)

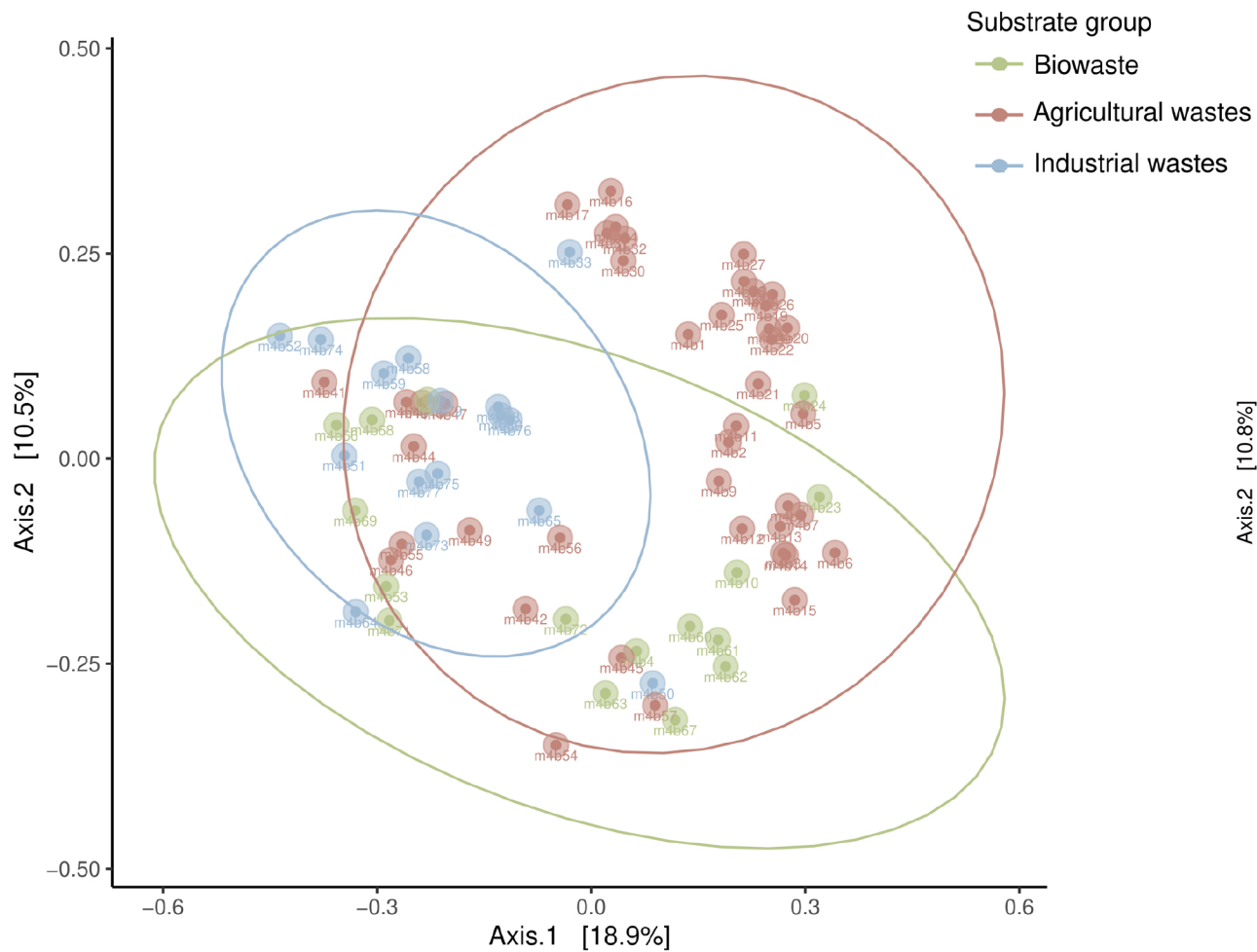

(B)

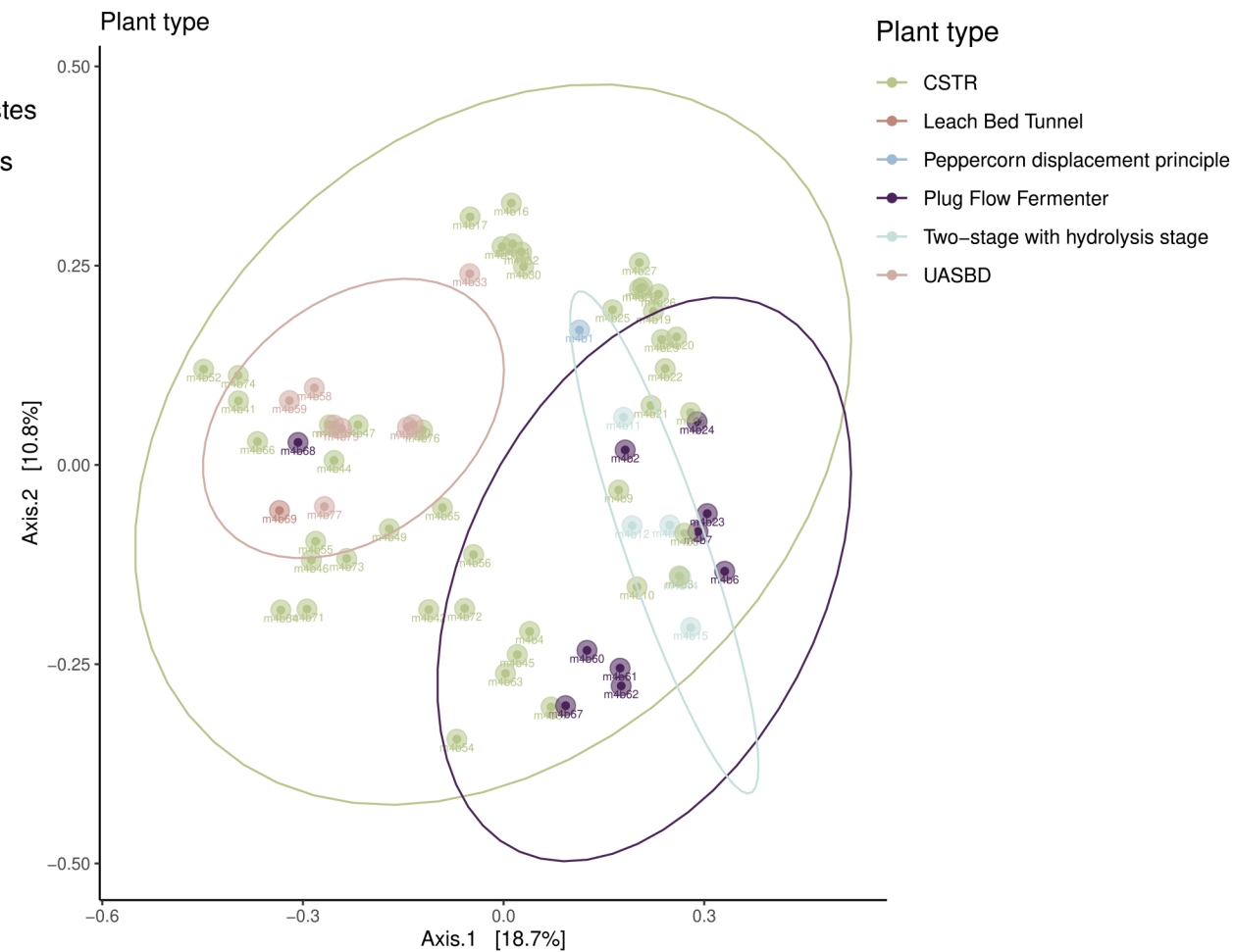

Supplement: Supplementary file 4 — Supplementary Figure 4. PCoA analysis of the three substrate groups (A) and different reactor types (B). The low p-value (p-value=0.001) obtained with the PERMANOVA test (Adonis2) shows that there is a significant difference between the groups. Both tests were performed in RStudio. [file 13068_2024_2525_MOESM4_ESM.pdf]

# Country comparison

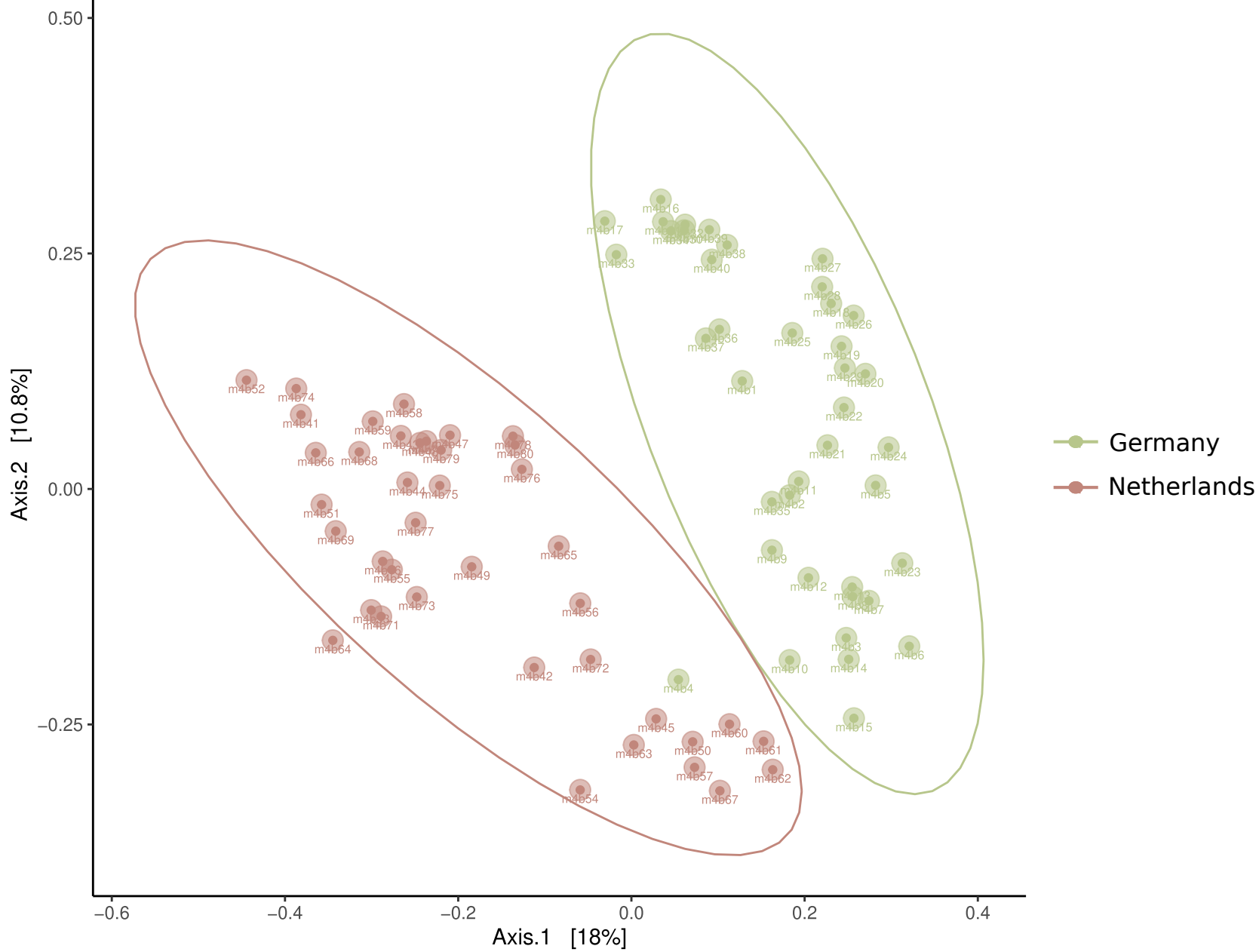

Supplement: Supplementary file 6 — Supplementary Figure 6. Most abundant genera in each country, discovering country-specific microbiomes. [file 13068_2024_2525_MOESM6_ESM.pdf]
