# Supplementary material for: Multivariate comparison of taxonomic, chemical and operational data from 80 different full-scale anaerobic digester-related systems
Source: Biotechnol Biofuels Bioprod. 2024 Jun 20;17:84. doi: 10.1186/s13068-024-02525-1 (PMC11191226; doi:10.1186/s13068-024-02525-1)
Supplement: Supplementary file 5 — Supplementary Figure 5. Most abundant genera in each reactor type. [file 13068_2024_2525_MOESM5_ESM.pdf]

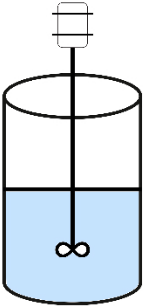

CSTR

- *Acetomicrobium*
- *Caldicoprobacter*
- *Syntrophaceticus*
- *Methanothermobacter*
- *Tepidimicrobium*
- *Echerichia-Shigella*

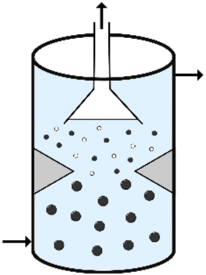

UASBD

- *Fastidiosipila*
- *Gallicola*
- *Christensenellaceae R7 group*
- *Methanosaeta*
- *Sedimentibacter*
- *Brooklawnia*

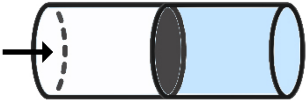

Plug flow fermenter

- *Brooklawnia*
- *Methanolinea*
- *Sphingomonas*
- *Paludibacter*
- *Coprothermobacter*

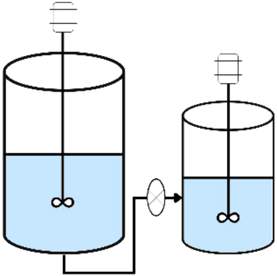

Two stage reactor

- *Brooklawnia*
- *Methanolinea*
- *Sphingomonas*
- *Paludibacter*
- *Coprothermobacter*
